# Cuba y seguridad sanitaria mundial: Cuba’s role in global health security

**DOI:** 10.1136/bmjgh-2019-002227

**Published:** 2020-05-13

**Authors:** Clare Wenham, Sonja K Kittelsen

**Affiliations:** 1 Department of Health Policy, London School of Economics and Political Science, London, UK; 2 Centre for Development and the Environment, University of Oslo, Oslo, Norway

**Keywords:** health policy, qualitative study

## Abstract

Cuba has been largely absent in academic and policy discourse on global health security, yet Cuba’s history of medical internationalism and its domestic health system have much to offer contemporary global health security debates. In this paper, we examine what we identify as key traits of Cuban health security, as they play out on both international and domestic fronts. We argue that Cuba demonstrates a strong health security capacity, both in terms of its health systems support and crisis response activities internationally, and its domestic disease control activities rooted in an integrated health system with a focus on universal healthcare. Health security in Cuba, however, also faces challenges. These concern Cuba’s visibility and participation in the broader global health security architecture, the social controls exercised by the state in managing disease threats in Cuban territory, and the resource constraints facing the island—in particular, the effects of the US embargo. While Cuba does not frame its disease control activities within the discourse of health security, we argue that the Cuban case demonstrates that it is possible to make strides to improve capacity for health security in resource-constrained settings. The successes and challenges facing health security in Cuba, moreover, provide points of reflection relevant to the pursuit of health security globally and are thus worth further consideration in broader health security discussions.

**Summary box**

- Debate on global health security has focused on how we collectively respond to outbreaks, including what role and responsibility states have in supporting response efforts to health crises beyond their borders, and in strengthening domestic public health capacities to detect and contain disease risks.
- Cuba’s history of medical internationalism and its domestic disease prevention and control activities speak directly to these debates; yet, Cuba is largely absent within global health security academic and policy discourse. Cuba’s international medical activities focused on routine health provision and crisis response, alongside its domestic disease control activities and universal health system demonstrate that Cuba has a strong health security capacity.
- Health security in Cuba also faces challenges, however, particularly with respect to the trade-off between civil liberties, regime preservation and security in protecting the state and its population from diseases threats, and with respect to resource constraints, exacerbated by the US embargo.
- Cuba does not frame its disease control activities within a discourse of health securitywhich impacts Cuba’s visibility and participation in the global health security regime. Cuba demonstrates that it is possible to make strides to improve capacity for health security in resource-constrained settings facing multiple challenges, although within a particular political context where social controls exercised by the state raise questions of civil liberties.
- The successes and challenges that characterise Cuba’s health security activities speak to broader global health security debates and are thus worth engaging with more explicitly in contemporary health security discourse and practice.

## Introduction

Global health security, defined here as global policy and activity to prevent, detect and respond to outbreaks of infectious disease, has emerged as dominant policy field in global health. Notable outbreaks of Severe Acute Respiratory Syndrome (SARS) (2002/2003), H1N1 (2009), Ebola (2014–2016; 2018–) and Zika (2016) have highlighted the mutual vulnerability to infectious disease and the need for a collective public health response. This has created an (almost) global policy path dependency focused on prevention, detection and response,^1 2^ exemplified by the International Health Regulations (2005), the defining piece of international law to manage the transborder spread of infectious disease, and the implementation of national health security strategies aimed at minimising the threat posed by pathogens to societies and economies.

The 2014–2016 Ebola epidemic in West Africa drew particular attention to how we collectively respond to outbreaks, including what role and responsibility states have in supporting response efforts to health crises that happen beyond their borders, and in strengthening domestic public health capacities to detect and contain disease risks, as articulated in Sustainable Development Goal (SDG) 3.D.^3^ In the wake of this epidemic, academic and policy debate has turned to how the system for global health security can be reformed.^4–6^ This has included a focus on how national and global capacities can be enhanced to rapidly respond to health emergencies and how we can connect global health security activities to broader health system strengthening efforts, including the movement towards universal health coverage (UHC), as championed by WHO Director-General Tedros Adhanom Ghebreyesus.^7^

Cuba’s activities in global health security speak directly to these debates. Indeed, Cuba has made considerable strides in infectious disease control, comparable to that of high-income countries, doing so in a low-income setting beleaguered by a struggling economy, and facing resource and access challenges from the US embargo. These activities are embedded within a strong integrated health system, of which a key constituent part is preventative medicine (including vaccination for infectious disease) and UHC free at the point of care. Through its work internationally, moreover, Cuba has moved towards supporting SDG 3.D by assisting other countries in meeting their core capacities for surveillance and response for disease control. Importantly for the global health audience, Cuba demonstrates that it is possible to make strides to improve capacity for health security in resource-constrained settings facing multiple competing challenges, although this has been in a context of an authoritarian state where civil liberties have been questioned.^8^

Given these achievements, it is interesting that Cuba has not been referenced in broader global health security debates; yet Cuba’s lack of visibility in health security academic and policy discourse is noticeable. This could be attributed to Cuba’s isolation geopolitically, as a small island state, and given that the health security policy field has traditionally been Western (US) dominated.^9^ The Cuban government may not have wanted to engage in this debate, or been able to participate, based on political tensions, ideological differences and/or the US blockade of the island. Alternatively, the geographical focus of health security discourse (and associated pragmatic efforts) has shifted temporally and geographically as the perception of ‘next big one’ has expanded from being focused solely on the threat of pandemic influenza in South-East Asia to include haemorrhagic fevers in low-income and middle-income countries (LMICs) in Africa. Cuba might have been absent from global health security narratives, similar to most of Latin America, because it had not been prey to a major epidemic that has put its health system surge capacity in the spotlight. However, with the spread of Zika across the Caribbean and to Cuba, despite the government’s denial of the outbreak,^10^ this may not be an adequate explanation. We suggest a third consideration for the lack of Cuban visibility in discussions of health security: that Cuba does in fact demonstrate key traits which we understand to be health security, but it does so in the name of solidarity and part of a socialist project which seeks to export the strengths of the Cuban regime internationally. We argue that health security is alive and well in Cuba and offers insights into how we might strengthen health security globally, particularly in LMICs which is vital to future preparedness efforts, although we consider these questions cautiously, recognising the particular political context in Cuba and the trade-offs of civil and political liberties which come at the costs of such a strengthened health security position.

To demonstrate our argument, we focus on what we consider the key traits of Cuban health security, conceptualised as falling into two main categories: Cuban medical engagements internationally, and Cuban health provision domestically. We then consider some of the challenges for health security in Cuba, notably the tension between providing complete security from the threat of disease and protecting civil rights within the state, and the impact of the US embargo on Cuban health security, before reflecting on what we can learn from the Cuban experience in improving health security globally.

This paper uses a case study methodology to understand how Cuba exhibits tendencies in global health security. The empirical material for this paper was drawn from a series of semistructured elite interviews conducted in Havana in December 2018 with policymakers, medical professionals and other infectious disease control experts working in Cuba. These data were subsequently transcribed, and thematic analysis was conducted to identify key trends that appeared in the qualitative material. We further undertook documentary analysis of policy documents from a range of sources, including the Ministry of Public Health (MINSAP), CubaCoopera (a government department responsible for medical internationalism) and the WHO/Pan American Health Organization (WHO/PAHO), along with media sources both from Cuba (*Granma*) and internationally. These documents were sourced both in English and in Spanish, and where necessary, translations are the authors’ own.

This material was triangulated against secondary literature including academic literature on health policy, international relations and Cuban/Latin American studies. These sources were identified through Medline, Google Scholar and snowballing from these and international and Cuban contacts. This latter step was important for two reasons: First, having undertaken interviews with policymakers from a one-party state that has worked hard to control information, we wanted to ensure that we were not solely reproducing the narrative that the Cuban government wished us to voice, but that this was contextualised amid other research and findings within health and/or security analysis of the island. Second, as Hollander states, short-term research visits to socialist countries can be wholly misrepresentative,^11^ so we wanted to ensure that our findings were reflective of broader debates and narratives within the literature on the Cuban health system.

## Cuba’s medical internationalism

Cuba’s programme of medical internationalism has been well documented by Latin-American scholars and anthropologists.^12–15^ Medical internationalism involves sending medical brigades on missions abroad to offer routine health services, respond to crises as well as bringing medical students and patients back to Cuba for education and/or treatment. We consider this to be the most apparent embodiment of Cuban health security activities internationally, where the state appears to be supporting response efforts for emerging pathogens by providing human resources and technical expertise within outbreak response, and health systems capacity-building elsewhere in the world, such as the training of international medical professionals.

Particularly significant from the standpoint of global health security is the role that these Cuban doctors, particularly the Henry Reeve Brigade, have played in supporting responses to a number of epidemic outbreaks. Brigades were deployed to El Salvador, Ecuador, Nicaragua and Honduras between 1999 and 2003 to support dengue fever response efforts.^12^ Moreover, the Cuban government was one of the first to respond to the 2014 Ebola outbreak in West Africa and its Henry Reeve Brigade constituted the largest international bilateral support to arrive in the region.^16^ This stands in contrast to the criticism that the global community as a whole has faced for its delayed and inadequate response to the crisis.^4^ The Cuban delegation, under WHO, was particularly involved in direct care to patients in Ebola Centre Modi (Liberia), Kerry Town and Port Loko (Sierra Leone) and supported the establishment of the Coyah Ebola Treatment Centre (Guinea). To do so, Cuban *brigadistas* received comprehensive training at the Tropical Medicine Institute, Instituto Pedro Kouri (Havana), demonstrating the significant health security competency and capacity within Cuba. This training included the use of personal protective equipment, biosecurity standards for patients and staff, treatment and diagnostic techniques, and risk scenario activity planning, and can now be used to launch a response to other diseases (Interview, Instituto de medicina tropical Pedro Kouri).

More routinely, Cuba deploys up to 20% of all its medical professionals to a number of non-crisis settings.^17^ This has involved medical teams totalling over 325 000 health personnel, in over 150 countries, including within the WHO and the G8 states.^18 19^ These Cuban medical teams have tended to work in rural communities or in urban slums, areas that often have not had state medical provision prior to Cuban arrival due either to an inability to recruit medical practitioners to live and work in these areas, or a lack of monetary motivation to establish provision where the local community has not been able to afford associated care costs, such as out-of-pocket payments. As routine access to health professionals facilitates the detection of diseases sooner, such activity can be considered to directly contribute to increased global health security, although notably this has not necessarily been the aim of any such brigade’s mission. Indeed, despite these efforts to support the development of health systems and respond to health crises, Cuba does not frame these activities within the discourse of health security. Rather, Cuba’s medical internationalism is framed as the embodiment of international solidarity and considered an extension of Cuba’s national health policy within the socialist framework. Interviewees cited ‘serving a global population’ rather than protecting Cuba’s citizens against pathogen spread as reason for crisis interventions.^9^

Recent media coverage of the closure of the *Mais Medicos* programme^20^ in Brazil has focused on the political determinants of these overseas medical missions, where Cuban doctors are sent to provide healthcare in exchange for oil or hard currency.^12 21^ Political motivations have also been suggested as standing behind Cuba’s provision of over half of the medical professionals for Mission *Barrio Adentro* in Venezuela, Chavez’s (now failing) health and social welfare programmes focused on delivering universal healthcare across poorer neighbourhoods.^22^ Such activities have spurred discussion on the motivation for this Cuban international activity, whether driven by international prestige,^14^ goodwill, soft power,^12^ solidarity^13^ or economic gain.^23^ Cuba’s medical missions have also been criticised for violating the (labour) rights of those deployed. There is some suggestion, for example, that these doctors may not have autonomous decision-making regarding whether they want to deploy on these international missions, but rather are forced to do so by the government.^24^ Recent legal proceedings in Brazil, moreover, have sought to show that the Cuban model of exporting physicians for economic gain violates the International Labour Organization’s agreement on the protection of wages.^24^ This is based on the findings from the *Mais Medicos* programme that suggest that Cuban medics received significantly less in wages than their international counterparts, with the majority of the wages paid directly by Brazil to Cuba for its doctors being retained by the Cuban state.^23 24^

Discussions about the profit-making nature of these overseas medical missions suggest that Cuba’s medical internationalism may be less about a notion of international solidarity or global health security, but rather functions as a form of income generation for the government within a failing economic system. In this respect, the contribution of these missions to global health security might be overegged and simply a by-product of government financing. Nevertheless, we suggest that this activity could be considered part of a Cuban contribution to global health security and may offer lessons for strengthening national and global rapid response capacities for disease emergence, as the deployments and training of the Henry Reeve Brigade suggests. Indeed, the global response to the 2014–2016 Ebola epidemic was criticised as being ‘hampered by a lack of trained and experienced personnel willing to deploy to the affected countries’.^25^ Aside from important concerns regarding the rights of medics deployed, Cuba’s medical brigades and the training they receive offer insights into how such a rapid response capacity might be achieved. Moreover, the focus of Cuba’s medical internationalism in strengthening the provision of routine health services in resource-constrained settings speaks to a broader vision of health security than disease containment and response alone—a focus of current debates concerning how global health security activities can be integrated in a more sustained way into broader health system strengthening and UHC aims. Yet, this deployment of Cuban teams has also raised concerns of taking jobs away from locally qualified physicians and healthcare workers, which raises further questions of sustainability within health systems^24^ as a cornerstone of UHC.

## Cuba’s integrated health system

Discussions on Cuban healthcare tend to focus on its government-run, integrated health system,^26^ rooted in a fundamental right to health as part of the socialist project; to provide a range of preventative and curative services free of charge to all. These services have produced world-leading health outcomes and demonstrate what is possible to accomplish in a resource-poor setting, allowing Cuba to demonstrate a strong domestic health security position, although the veracity of some of these claims has been disputed.^27^ In particular, critiques have highlighted that the Cuban image of a high-performing system appears to be almost exclusively based on infant mortality rates—an explicit point of focus of Cuban government policy—at the cost of other health indicators, including maternal mortality, disability, disease exposure and life expectancy, and underinvestment in the social determinants of health more broadly.^27 28^ Moreover, even this infant mortality rate has been questioned^29^ with regards to its accuracy, relative to the counterfactual^30^ and potential case-definitional differences to routine practice making Cuba dramatically outperform its regional (and global) neighbours.

Focusing on infectious disease in particular, there are three key components to Cuba’s domestic health security: disease surveillance and control, Cuba’s biotechnology industry and universal healthcare. First, Cuba has focused considerable efforts on reducing or eliminating disease threats from its territory. This has included decades of work reducing HIV/AIDS infection, including eradicating mother-to-child transmission of HIV, through prevention and destigmatisation campaigns,^31^ although this has come at a cost of human rights through the compulsory testing and indeed quarantining of those infected (Interview, Biosecurity expert).^32 33^ Similarly, Cuba eliminated malaria on the island in 1973, and maintains this status, although through mandatory prophylaxis of those returning (or indeed arriving) in Cuba from endemic regions. The country has also managed to eliminate 13 diseases since 1960, including measles, rubella, mumps, tetanus, meningitis B and C, and hepatitis B. (Interview, Ministerio de Salud Publica).

When the threat of Zika spread across the Americas, Cuba was quick to take proactive moves to stop the introduction of the disease within its borders. This included mass fumigation campaigns, the deployment of the military to clean civic spaces where mosquitoes may grow, and an appeal to citizens enforced by law, to support control efforts and ensure that private dwellings remained clear from mosquitoes and therefore, disease.^34 35^ Such procedures are rooted in disease-specific action groups for health concerns; for example a dengue task force, empowered at multiple levels of governance from the municipality to the MINSAP and deployed in the case of a dengue outbreak with the exclusive task of bringing about the outbreak’s end (Interview, Pan American Health Organization (PAHO)). This provides Cuba with surge capacity to systematically respond to and manage health security concerns that occur within its borders. These groups work alongside the routine health service, and with civil defence units to respond to crisis events and mobilise quickly to minimise any potential threat to the population (Interview, Pan American Health Organization (PAHO)).

More systematically, Cuba has a distinct focus on rigorous point-of-entry controls. Indeed, the main role of MINSAP’s health surveillance unit is to prevent the introduction of infections to Cuba (Interview, Ministerio de Salud Publica). When Cuban nationals return to Cuba from states that have endemic infectious diseases, they are subject to rigorous airport screening. This information is passed to their neighbourhood doctor for daily or weekly follow-up to ensure that individuals are not experiencing unusual symptoms and if they are, to facilitate rapid treatment, including if necessary, mandatory treatment and/or quarantine to avoid further disease transmission (Interview, Instituto de Medicina Tropical Pedro Kouri). The ability of the health system to facilitate such personalised follow-up, to ensure rapid treatment and to limit further infection is remarkable, and from a public health perspective, it far outperforms any health system in the global North, if not from a human rights perspective. These point-of-entry protocols are enforced for every flight and include airport scanners and in-airport medical professionals. If necessary, those arriving in Cuba may be taken directly to a doctor and/or the tropical medicine institute, IPK (Interview, Instituto de Medicina Tropical Pedro Kouri). This quarantine and prophylaxis procedure at Instituto de Medicina Tropical Pedro Kouri (IPK) includes all medics returning to Cuba from Ebola-infected or malaria-infected locations, who are subject to a mandatory 21 days stay at IPK.

Second, Cuba has a booming biotechnology industry, with significant investment made by the Cuban state into research and drug development to ensure access to medicines should an outbreak emerge. As Cuba is unable to purchase many global biomedical products, owing to the strict embargo placed on the country by USA, the strides taken to ensure indigenous production of these medicines, treatments and future research represents demonstrable activity in maintaining Cuba’s health security to ensure a healthy and productive population. For example, this has included development of a vaccine for dengue fever, meningitis B, hepatitis B and many more besides.^36 37^ Moreover, a biosecurity level 3 laboratory (Interview, Ministerio de Salud Publica) and the development of other biomolecular diagnostic laboratories that can do diagnosis incountry mean that Cuba does not have to send virus samples elsewhere (Interview, Ministerio de Salud Publica).

Third, Cuba maintains almost UHC. UHC has been championed as a key mechanism to developing health systems resilient to disease outbreaks.^38 39^ Cuba demonstrates how the mechanisms and processes for providing UHC to the population also provide the foundation for effective infectious disease control. Cubans can seek medical advice through free and easily accessible consultations with health professionals through the *consultorio* (neighbourhood health clinic), established ‘on every block’ with a doctor and nurse in each community—with approximately one *consultorio* per 1000 people. This has only been possible through significant government investment in healthcare as a priority since the Revolution (although the motives for this are in part because the majority of doctors in Cuba fled at that time (Interview, Pan American Health Organization (PAHO)).

For more specialist care, patients are referred to a *policlinico* (polyclinic), particularly specialising in maternal and child health, chronic disease support and minor surgeries. The initial aim of this family centred programme was to decentralise hospital activities, to free up space in the system to manage the most serious of cases and to allow for surge capacity in the event of an emergency. Yet the medics, and importantly nurses, who work in these *consultorios* and *policlinicos* who offer general medical provision also play a decisive role in epidemiology and disease prevention. In terms of epidemiological surveillance, the ‘doctor on the corner’ functions to record any epidemiological changes in the community. For example, dengue outbreaks, which occur with some frequency across Cuba, are rapidly detected and within a matter of days integrated vector control activities will have begun due to the epidemiological reporting of the local *consultorio*. The close contact between the nuclear family and the doctor on every corner facilitates this rapid detection and response, ensuring that infections do not become major outbreaks and that infection control protocols are implemented (Interview, Ministerio de Salud Publica). This is in spite of no automated infrastructure and a paper-based surveillance system in a resource-poor setting.

This infectious disease surveillance also pre-empts disease outbreaks through annual health checks on all members of the community (*dispensarizacion*). Through this process, the *consultorio* preclassifies potential ‘at risk’ citizens based on social factors which may make them predisposed to infectious disease should it arise (Interview, Ministerio de Salud Publica; Interview, Pan American Health Organization (PAHO)). This preventative approach to disease control also relies on preventative care through increased vaccination coverage and health awareness among the population, reducing the chances of an outbreak taking hold in the country, the latter evidenced by the successful management of the HIV/AIDS crisis on the island.^31^ Thus, Cuba demonstrates how the mechanisms and processes for providing UHC to the population also provide the foundation for effective infectious disease control, although this has been in a political context where the components of disease surveillance, biotechnology and universal healthcare form part of a broader narrative of regime preservation. Indeed, if the goal of the Cuban state is to preserve its borders and political narrative, then ensuring that its population has protection from infectious disease and access to routine healthcare functions also to reassure citizens in an effort to avoid internal sedition and lack of faith in the socialist project. Nevertheless, while learnings from Cuba must be considered within an instrumentalist paradigm and recognise that Cuba’s health security activities may not be as portable to different political and social environments, Cuba’s integration of community-based healthcare and disease surveillance and control activities does provide a pertinent example for broader global health security/UHC debates as to what this might look like in practice, and how effective UHC can be for ensuring global health security.

## Cuban health security: challenges and limitations

Despite Cuba’s strong health security position, Cuban health security activity is not framed as such, and this framing may well have an impact on Cuban visibility within this global policy space. In turn, there are further impediments to Cuban participation and recognition as a leader within the global health security architecture. For example, Cuba claims to have met all the core capacity requirements for disease surveillance and control under the International Health Regulations (IHR) (2005), but has yet to agree to undergo a joint external evaluation (JEE) to externally verify these capacities. Although interviewees stated that plans were afoot for a JEE in 2019, we were unable to find evidence of this at the time of writing. This lack of clarification over the status of the JEE is also mirrored in Cuba’s failure to report outbreak events to WHO, as required under the IHR (2005), including during the Zika outbreak and rumoured cholera outbreaks across the island. Where reports have been made, the case numbers reported have sometimes been manipulated so as not to reveal the full extent of the outbreak (Interview, Pan American Health Organization (PAHO)). Thus, while Cuba may demonstrate achievements in global health security, it also appears to be a reluctant player in the global health security regime.^40^

Cuban health security activity, moreover, is not without its own challenges. As highlighted above, the ability for the state to mandate prophylaxis or quarantine for those potentially infected by a particular virus, not to mention pre-emptive activity on citizens deemed to represent risks to collective health security on the island, raises questions of civil liberties and human rights within the Cuban health sector. Much has been written about Cuba’s abuse of human rights and tight authoritarian control in other sectors,^41 42^ so this may not be of surprise from a broader Cuban structural perspective. Yet, within the health sector, there is no right to privacy in the physician-patient relationship, no right to informed consent and no right to refuse treatment.^43^ These challenges point to an inherent tension within health security between the protection of civil liberties and public health, as encapsulated by the Siracusa Principles.^44^ The social control exercised by the Cuban state thus raises questions of where the line between liberty and security lies and what rights governments have to restrict individual freedom in the name of public health—a debate that has also played out in other contexts, such as in the handling of patients with multidrug-resistant tuberculosis globally.^45^

A further challenge to Cuba’s health security is the level of underdevelopment that remains across much of the island. Vast swathes of the island live in poverty, without routine access to water or sanitation services, with open sewers or water that is unable to drain because of poor civic planning (Interview, Pan American Health Organization (PAHO)). This can have a direct influence on susceptibility to vectorborne or waterborne disease. Failure to maintain sewage systems in a civic infrastructure struggling to financially support itself has led to an increase in diarrhoeal disease and cholera.^16^ This lack of investment in the socioeconomic determinants of health, potentially as a consequence of the decisive focus on infant mortality within the health sector, poses a contradiction to so much of the success that Cuba can show in terms of an exemplary health security capacity in such a resource-poor setting. It also demonstrates the inequalities across the system; while the *consultorio* model and thus ‘health security’ may work in urban areas, this may not be replicated across the system in poorer zones (Interview, Pan American Health Organization (PAHO)).^46^ Thus, socioeconomic development and indeed, resources remain a key limitation of health security in Cuba. Lack of government funds means that in the event of a major outbreak there may be limitations on equipment, deployment activities elsewhere, and research and development achievements.

These resource constraints are exacerbated by the US embargo, which has been almost continuously in place since 1960. This has wide-ranging effects across areas of domestic policy, and despite having little impact on life expectancy,^30^ we propose that the embargo does have a potential impact on the health security strengths of the system.^47^ For example, the embargo on US goods means that pharmaceutical ingredients or products produced in USA or with a patent held by a US company are not available in Cuba. This means that Cuban access to newer international drugs is limited and may directly impact on the health security of the nation if a newly emerging pathogen appears and a drug is developed that Cubans are not able to access (Interview, Ministerio de Salud Publica). This also extends to medical equipment. There have been efforts by Cuban medics to circumvent this medical embargo, through sourcing medication from third party states, but this has been met with indignation and legal challenge by the USA.

## Conclusion

While it is impossible to imagine a replication of the Cuban health system in other resource-poor settings owing to the uniqueness of the political situation, this is not to say that meaningful lessons cannot be ported. In particular, Cuba demonstrates how a health system that is prevention-oriented and community-based contributes to reducing infectious disease threats—a key point of focus in contemporary global health security debates. Here Cuba provides a much needed example of how disease surveillance and control efforts can be integrated with community-based primary healthcare to provide for health security in low-income settings. Cuba’s medical internationalism, moreover, demonstrates the effectiveness of frequently offering national doctors for international disease control efforts, allowing for a well-trained and readily deployable medical corps to respond to disease both within Cuba and abroad. The training provided to the Henry Reeve Brigade in responding to the 2014 Ebola outbreak is particularly illustrative of how national and global surge capacity to respond rapidly to health crises might be achieved. The focus on strengthening routine healthcare as well as health crisis response in Cuba’s medical internationalism, moreover, opens up space to reflect on how global health security activities might be integrated into longer-term health interventions.

Despite these activities, however, Cuba remains largely at the margins of global health security policy and debate, with implications for Cuba’s engagement with the global health security regime, as encapsulated by the IHR (2005), and for Cuba’s visibility in global health security policy and practice. Indeed, the US embargo, Cuba’s development and resource challenges, and the tight social controls exercised by the state in managing disease threats in its territory also speak to broader global health security issues. These concern global access to essential medicines and commodities, the development of sectors beyond health alone in reducing vulnerability to disease, and the trade-off between civil liberties and public health in disease control efforts. The Cuban case thus offers opportunity for more in-depth reflection as to the strengths and weaknesses of contemporary health security practices, although with the caveat that lesson learning should be applied cautiously in this context given the tensions within the Cuban political system in relation to civil and political freedoms, the critiques concerning Cuba’s instrumentalist promotion of its health system and the trade-offs which had been decisively made for the prioritisation of disease control and infant mortality.
